# Efficacy of pedagogical framework in neonatal resuscitation skill learning in a resource-limited setting: a randomized controlled trial

**DOI:** 10.1186/s12909-021-02846-x

**Published:** 2021-08-18

**Authors:** Mishal Liaqat, Muhammad Hussain, Muhammad Afzal, Maryam Altaf, Sadia Khan, Syed Amir Gilani, Iram Liaqat

**Affiliations:** 1 Lahore School of Nursing, The University of Lahore, Lahore, Pakistan; 2 Faculty of Allied Health Sciences, The University of Lahore, Lahore, Pakistan; 3 Department of Zoology, The Government College University, Lahore, Pakistan

**Keywords:** Neonatal resuscitation, Education, Pedagogy, Nursing, Students, Skill

## Abstract

**Background:**

The educational efficacy in neonatal resuscitation relies on the subject and teaching strategies. Therefore, it is imperative to test diverse educational methods if they are more instructive to engage students in active learning and practicing knowledge. Hence, the present study aims to investigate the efficacy of a pedagogical framework in neonatal resuscitation skill learning among nursing students in a resource-limited setting.

**Methods:**

A single-blind randomized controlled trial was conducted between October 2020 to March 2021. Sixty nursing students in the 3rd and 4th year of professional training were randomly allocated to the pedagogy and the traditional group. The pedagogy group learned via 6-step LSPPDM (Learn, See, Practice, Prove, Do, Maintain) pedagogy including lectures, video, clinical observation, skill sessions under supervision, and self-directed practice. The traditional group learned through 2-step (Learn, Practice) method that included lectures and skill sessions under supervision. The outcomes measured included technical and non-technical skills in neonatal resuscitation. The technical skill deals with steps such as stimulation, ventilation, oxygenation, intubation, chest compression, medications, and reporting. Non-technical skills refer to teamwork skills that focus on the interaction between leader and helper. Both skills were measured through previously published validated tools two times before and after the intervention by blinded assessors in a simulated delivery room.

**Results:**

Overall, the skill was significantly improved in both groups after intervention. Yet, the results showed that the mean difference of technical skill score in the pedagogy group (24.3 ± 3.5) was significantly higher (*p* <  0.001) compared to the traditional group (16.2 ± 2.4). Likewise, the mean difference of non-technical skill score in the pedagogy (36.9 ± 1.9) was highly significant (*p* <  0.001) compared to the traditional group (31.2 ± 1.7).

**Conclusions:**

The LSPPDM pedagogy was found more effective in enhancing technical and non-technical skills in neonatal resuscitation compared to the traditional method. The results of this study support the efficacy of the 6-step LSPPDM pedagogy in the education of nursing students regarding neonatal resuscitation in a resource-limited setting.

**Trial registration:**

Prospectively registered at ClinicalTrials.gov (NCT04748341).

**Supplementary Information:**

The online version contains supplementary material available at 10.1186/s12909-021-02846-x.

## Introduction

Globally, 136 million births are reported annually. About, 4 million births suffer asphyxia [1] in which 10% entail simple resuscitative efforts while 0.1% need advanced measures to initiate breathing [2]. Despite all the efforts, approximately one-quarter of all neonatal deaths occur due to asphyxia [3, 4]. Unfortunately, 98% of these deaths occur in low, and middle-income countries [5], among which Pakistan is ranked as number three on top 10 countries with the highest neonatal deaths [6]. It is suggested that effective resuscitation at birth can prevent a major proportion of these deaths [7] in which education plays an integral role.

The first educational program in neonatal resuscitation was initiated in 1987 in the United States [8] but was quite challenging to implement in low-resource settings. Thus, Helping Babies Breathe (HBB) was designed especially for developing regions. The elements most pertinent to HBB are the emphasis on active learning with skills demonstration, practice, and clinical scenarios that incorporate decision-making and communication skills among the participants [9]. Singhal et al. [10] reported the educational validation of HBB in neonatal resuscitation among health care providers’ knowledge, skill, and self-efficacy in two resource-limited areas; Kenya and Pakistan. Since 2010, it has been adapted in more than 80 countries and contributed essentially to improving quality care [11].

Pediatric care is a basic component of the nursing curriculum, instituting resuscitation as a vital part of nursing students’ skills during their undergraduate studies [12]. Yet, the vast majority of nursing students are unprepared and lack confidence in basic lifesaving skills such as resuscitation [13]. Similar to many developing countries, Pakistan also prefers the traditional method of medical education [14]. Traditional education of neonatal resuscitation based on didactic lectures followed by a practice on manikins [15, 16] is prevalent in all nursing schools of Pakistan. Previously, several studies compared traditional methods of education by integrating new strategies and concluded that new methods are superior to the traditional methods [17, 18]. Hence, it’s imperative to test traditional methods with diverse educational strategies and to figure out which one is more instructive among nursing students, particularly in resource-limited settings.

The “Learn, See, Practice, Prove, Do, and Maintain” (LSPPDM) pedagogy is one of the frameworks synthesized after intensely reviewing the literature. This framework is acting as a guiding path in teaching and learning of procedural skills [19]. It exposes the learners in two phases; one is cognitive and the other is psychomotor. In the cognitive phase, the learner meets the two steps of “Learn” and “See”. The first step “Learn” is focusing on acquainting knowledge through didactic lectures. It progresses to the second step “See” based on visual learning through procedural videos [20]. Whereas, the psychomotor phase deals with the steps of “Practice”, “Prove”, “Do” and “maintain”. In the “Practice” step learners deliberately perform the learned skill in the simulation environment. In the fourth step “Prove” the learner’s skill was assessed on a simulator to prove proficiency [19]. The fifth phase “DO” exposes the learner to perform the skill in the clinical setting under supervision. Then, finally the sixth phase “Maintain” the learner further practices the skill in the clinical area supplemented with simulation as needed [20]. Thus, a step-by-step process is facilitated by the instructor.

To the best of the researcher’s knowledge, no published literature was found on the efficacy of a pedagogical framework in neonatal resuscitation skill learning among nursing students of Pakistan. In addition, the decline in skill is more reported than knowledge which further necessitates that a focus on improving skill is crucial. The current study was planned to measure skills in terms of technical and non-technical skills [21]. Technical skill is defined as the competency in each step from the technical aspect [22] and includes all essential steps of resuscitation such as stimulation, ventilation, oxygenation, intubation, chest compression, medications, and reporting [23]. Non-technical skills refer to teamwork skills that focus on the interaction between leader and helper [24] and include task management, team working, and situation awareness. Both technical and non-technical skills complement each other in the efficient practice of the intervention and improving clinical outcomes [25].

## Objective

The objective of the study was to compare the technical and non-technical skills among undergraduate nursing students learning neonatal resuscitation through “Learn, See, Practice, Prove, Do, Maintain (LSPPDM) pedagogy” compared to those who had learned through the traditional method.

## Methods

### Study design and setting

**Fig. 1:**
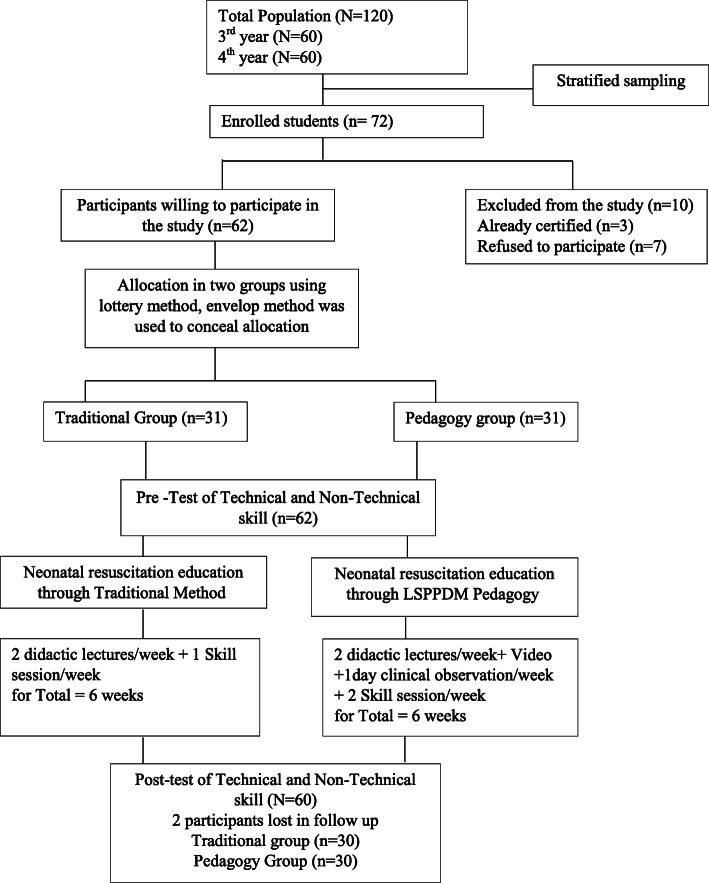
Consort Diagram

The study was a single-blind randomized control trial and adhered to the CONSORT guiding principle (Fig. 1), conducted between October 2020 and March 2021. The research design was in compliance with the guidelines and regulations of the Declaration of Helsinki Ethical Principles for Medical Research involving human subjects and was approved by the Institutional review board of the University of Lahore via reference number IRB-UOL-FAHS/775/2020. The study was prospectively registered at ClinicalTrials.gov (dated:10/02/2021) via reference number: NCT04748341.

The setting was the Nursing department of Allama Iqbal Medical College, Lahore Pakistan, a specialized tertiary level educational center in the public sector that offers (2 & 4 year) graduation in nursing and 1-year post basic specialization in various disciplines. The participants were 3rd and 4th professional B.Sc Nursing students (4 years) enrolled in the current academic year of 2020–2021.

### Sample and sampling technique

The sample size was calculated using the mean and standard deviation of skill values from a previously published study [17]. The calculated sample size was 24 using the *OpenEpi* software having an 80% power at α = 0.05. This sample size was small to perform the statistical test with good efficacy. So, 30 in each group were taken and after adding a 20% dropout rate [17] the final sample size was 72 (36 in each group). The list of all nursing students of the 3rd and 4th year of professional training was developed and the participants were recruited through stratified sampling by year of education (3rd or 4th year). The study included the nursing students of 3rd and 4th year, enrolled in the Bachelor of Science in Nursing (4 years program). All students of 18–25 years of age gave informed consent. The nursing students who had already received any educational training on neonatal resuscitation and working as nursing assistants were excluded from the study. Initially, 62 participants (31 in each group) meet the inclusion criteria. Two participants were dropped in the follow-up for personal reasons, one didn’t attend the full course of intervention and the other was absent during the days of evaluation, hence yielded a total of 60 participants with 30 in each group.

### Randomization and blinding

The selected students were allocated in pedagogy and traditional group through the lottery method [26]. Allocation of participants was concealed using the envelope method. The facilitators, who assessed the student’s skills, remained uninformed regarding the student’s allocation status and sequence of the scenario in the pre-and post-assessment.

### Educational intervention

Six weeks of educational intervention were given to both groups. The material was taught in English. All the nursing students had 12-year schooling, where English was being used as a medium of learning in their previous education as well as in nursing. Furthermore, all medical and nursing education is in the English medium in Pakistan. Therefore, students faced no problem in understanding the study material in English. In addition, the instructor also explained the material in the local language (Urdu) to both groups for in-depth understanding.

#### Traditional method of neonatal resuscitation (traditional group)

The traditional group learned through the 2-step traditional method that included 2 didactic lectures/ week leading towards the second step of 1 skill session/week for 1 h under instructor supervision. Both the instructor and assessors were certified in Neonatal resuscitation and had more than 10-years of experience in the respective field. The lecture content was the same for both groups and was prepared from the Textbook of Neonatal Resuscitation, 7th edition of the American Academy of Pediatrics [27]. The lecture’s content was based on foundations, preparation, initial steps, positive pressure ventilation, endotracheal intubation, chest compression, medication, post-resuscitation care, and ethics in resuscitation and was presented in English. The students practiced their skills under instructor supervision on the manikins in groups of 4–5 in each group. The performance error was corrected immediately.

#### LSPPDM pedagogy method of neonatal resuscitation learning (pedagogy group)

The pedagogy group was taught through the 6-steps LSPPDM pedagogy steps including 2 lectures/week + video+ 2 skill sessions/week for 1 h. In the 1st step (learn) didactic lectures were given to the students leading to the 2nd step (video watch), a 30 min video on neonatal resuscitation (sss). The students first saw the video in the class and then the video was shared on their mailing address for frequent watching. In the 3rd step (practice), students practiced their skills on a low fidelity simulator under instructor supervision. The errors were corrected at the spot. In the 4th step (prove) students proved their skill through the checklist to enhance their learning and memory. In the 5th step (do) students observed neonatal resuscitation in real-life situations through the clinical rotation. Finally, the 6th step (maintain) was established through an additional one-hour skill session/week for a total of 6 h under self-directed learning. The instructor distributed 4–5 students per group for the self-practice session. The students further identified their own needs, areas of deficiencies and practiced accordingly on a scheduled day and time in the simulation lab.

**Fig. 2:**
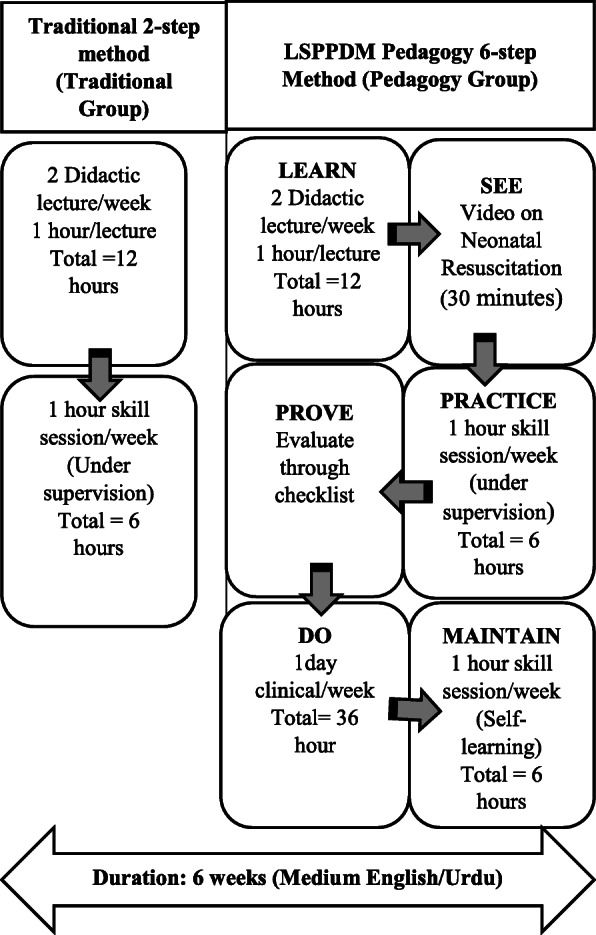
Comparison of the intervention between the Traditional and Pedagogy group

The comparison of intervention among both groups is given in Fig. 2.

### Methods for collection of data

Two evaluators were trained regarding the score criteria of each scale in a two-hour workshop before data collection [28]. The data were collected at two time points (before and after the intervention) in the simulation lab. The students were assigned code to mask their participation and blind data analysis.

### Evaluation environment

On the day of evaluation, students were divided into pairs. Each pair performed in two scenarios; in which one was acting as a leader and the other was the helper and vice versa [24]. The procedure was classified into assessment, preparation, initial newborn care, positive pressure ventilation, intubation, chest compressions medications, and reporting. All the simulation scenarios were performed in a simulated delivery room situation in the presence of two student facilitators. One was acting like a pregnant lady while the other was performing the role of a delivery nurse and presented the simulation scenario in a standardized fashion to each participant. The same scenario was used for the baseline and final assessments of both groups (see supplementary data) and was prepared from the Textbook of Neonatal Resuscitation, 7th edition of the American Academy of Pediatrics [27].

The student was scored only if students performed the step within an appropriate time and manner. All the students were allotted a maximum time of 10 min to perform all the steps of resuscitation. However, students were able to finish earlier than 10 min if all steps were followed correctly. A lab assistant was making a video recording of all students’ performance to validate the observations and scoring. Both evaluators simultaneously assessed and scored each student. Any discrepancy in scoring was resolved through reviewing the video to reach a consensus by both reviewers. For the maintenance of confidentiality, the videos were deleted after corroboration [29].

### Technical skill and non-technical skill tool

Neonatal resuscitation technical and non-technical skills were measured through a previously published validating tool [23]. The Technical skill tool contains a 30-item neonatal resuscitation technical skill checklist. Each of the correct actions was graded as 1 = yes and for wrong action 0 = no. The non-technical skills were measured through a 9-item checklist on a 5-point Likert scale. The technical and non-technical skill Cronbach’s α of 0.719, 0.938 respectively. While the intra-rater reliability of 0.885 for technical and 0.963 for non-technical skill was reported [23].

### Data analysis

The latest version of the Statistical Package for the Social Sciences (SPSS) 25 was used for data analysis. Descriptive statistics were used to express the sample’s characteristics. Both groups were tested for homogeneity in general and resuscitation specific characteristics using the χ2 -test, and t-test. To compare the difference between the two groups, an independent sample t-test was used. Paired t-test was used to compare the pre and post-test scores in both study groups. A *p*-value ≤0.05 was taken as significant.

## Results

### Homogeneity of the study participants

Sixty-two nursing students from3^rd^ and 4th year of professional training were enrolled and randomly allocated in the traditional and pedagogy groups through the lottery method. One participant from each group was dropped in the follow-up, thus a total of 60 participants were included in the final analysis. The mean age was 22.3 ± 0.75 years in the traditional group and 21.9 ± 1.1 years in the pedagogy group. In both groups, 29 participants (96.7%) had Urdu as a primary language and only 1 participant (3.3%) had Punjabi as the primary language. In the traditional group, 3 participants (10.0%) were from the rural area while in the pedagogy group, only 2 participants (6.7%) were from the rural area. In the traditional group, 28 participants (93.3%) had first division (> 60% marks) in the previous year’s examination and 2 (6.7%) had 2nd division (< 60% marks). while in the pedagogy group, all participants (100%) had the first division. In the traditional group, 5 participants (16.7%) had previous neonatal resuscitation exposure and 25 (83.3%) had no such exposure while 3 participants (10.0%) had previous neonatal resuscitation exposure and 27 (90.0%) had any such exposure in pedagogy group. The pre-education homogeneity between both groups was tested through Chi-square and t-test (Table 1).

**Table 1:** Homogeneity in demographic variables among participants’

Variables	Categories	Traditional Group	Pedagogy Group	***p***-value
Age (years)		22.3 ± 0.75	21.9 ± 1.1	0.075
Year of Professional Education	3rd	14 (46.7%)	15 (50.0%)	0.796
	4th	16 (53.3%)	15 (50.0%)	
Primary Language	Urdu	29 (96.7%)	29 (96.7%)	–
	Punjabi	1 (3.3%)	1 (3.3%)	
Residential Area	Rural	3 (10.0%)	2 (6.7%)	> 0.999
	Urban	27 (90.0%)	28 (93.3%)	
Division in previous results	First (> 60%)	28 (93.3%)	30 (100.0%)	0.492
	Second (< 60%)	2 (6.7%)	0 (0.0%)	
Previous neonatal resuscitation exposure	Yes	5 (16.7%)	3 (10.0%)	0.706
	No	25 (83.3%)	27 (90.0%)	

Table 2 shows neonatal resuscitation technical skill scores following a pre and post-test in both groups. A paired sample t-test was used to compare pre and post-test differences in groups. Overall, the technical performance was significantly improved (*p* <  0.001) in both groups after receiving education. An independent sample t-test was used to compare the mean change in technical skill scores between the two groups. Results showed that the mean difference in post technical skill score of the pedagogy group (24.3 ± 3.5) was significantly higher (*p* <  0.001) compared to the traditional group (16.2 ± 2.4). The pre-test non-technical skill score of both groups was similar. The post non-technical skill score of the traditional and pedagogy group was 31.2 ± 1.7 and 36.9 ± 1.9, respectively. Likewise, the mean difference in non-technical scores was significantly higher (< 0.001) in the pedagogy group compared to the traditional group.

**Table 2:** showing the comparison of mean technical skill scores between both groups

Variables	Group	Pre	***p***-value	Post	***p***-value	Difference	***p***-value
Skill score	Traditional	3.30 ± 1.18	0.821	16.2 ± 2.4	< 0.001	12.9 ± 2.14	< 0.001
	Pedagogy	3.37 ± 1.10		24.3 ± 3.5		20.9 ± 3.1	
Non-Technical score	Traditional	23.2 ± 1.1	0.285	31.2 ± 1.7	< 0.001	8.0 ± 1.6	< 0.001
	Pedagogy	23.6 ± 1.7		36.9 ± 1.9		13.4 ± 2.0	

The Independent sample t-test was used to compare the post mean score of non-technical skills between both groups. Overall, the pedagogy group showed a higher score on each step compared to the traditional group. The post mean score of non-technical skills of the pedagogy group was found significantly higher for the first seven skills compared to the traditional group. While an insignificant difference was observed for two skills i.e., “vigilance and anticipation” and “Adequate medical knowledge”. Thus, the LSPPDM pedagogy method was effective in enhancing non-technical skills in neonatal resuscitation among nursing students (Table 3).

**Table 3:** Comparison of post non-technical skill score between both groups

S. No	Non-technical Skills	Traditional Mean ± SD	Pedagogy Mean ± SD	***p***-value
1	Recognizing the situation without delay	3.13 ± 0.73	3.97 ± 0.72	< 0.001*
2	Continuous evaluation of the patient	3.60 ± 0.62	4.20 ± 0.76	0.001*
3	Prioritizing problems, supporting others	2.80 ± 0.61	3.70 ± 0.60	< 0.001*
4	Following the algorithm	3.40 ± 0.50	4.33 ± 0.55	< 0.001*
5	Uninterrupted plans to act	2.93 ± 0.58	3.40 ± 0.50	0.002*
6	Leadership, coordinating activities	3.43 ± 0.68	4.00 ± 0.74	0.003*
7	Communication	3.77 ± 0.73	4.27 ± 0.58	0.004*
8	Vigilance and anticipation	3.90 ± 0.66	4.20 ± 0.61	0.073
9	Adequate medical knowledge	4.43 ± 0.50	4.60 ± 0.50	0.203

*Significant

The Chi-square test was used to compare the proportion of correctly performed skills between both groups. Overall, the pedagogy group showed a higher score on each step compared to the traditional group except for question number 29, where the pedagogy group score was lower (76.7%) compared to the traditional group (83.3%). While pedagogy group proportion of correctly performed skills were found significantly higher for skill question number 6, 7, 9, 10, 12, 13, 15, 17, 19, 20, 21, 23, 24, 25, 26, 27, and 28 compared to traditional group. An insignificant difference was observed for the remaining skills (Table 4).

**Table 4:** Comparison of post correctly performed skills between both groups

S. No	Skills	Traditional n (%)	Pedagogy n (%)	***p***-value
1	Checks sizes of suction catheters available and if suction equipment is working	18 (60.0%)	24 (80.0%)	0.091
2	Dries the baby and throws wet linen away	14 (46.7%)	20 (66.7%)	0.118
3	Checks pulse by palpation or auscultation	25 (83.3%)	28 (93.3%)	0.424
4	Stimulates the baby by knocking soles of the feet and rubbing the back	27 (90.0%)	30 (100%)	0.237
5	Suctions the baby first through the mouth and then through the nose	24 (80.0%)	29 (96.7%)	0.044
6	Places the head in correct position (nose is at the highest point)	22 (73.3%)	28 (93.3%)	0.038*
7	Takes a proper sized mask	15 (50.0%)	28 (93.3%)	< 0.001*
8	Starts to ventilate	29 (96.7%)	30 (100%)	> 0.999
9	Checks that the mask is not leaking	9 (30.0%)	23 (76.7%)	< 0.001*
10	Check’s chest movements	9 (30.0%)	20 (66.7%)	0.004*
11	Does not over-expand the lungs	26 (86.7%)	30 (100%)	0.112
12	Maintains right ventilation frequency	24 (80.0%)	30 (100%)	0.024*
13	Maintains correct ventilation volume during resuscitation	10 (33.3%)	23 (76.7%)	0.001*
14	Uses air at the start	15 (50.0%)	22 (73.3%)	0.063
15	Notice’s cyanosis of the baby	8 (26.7%)	16 (53.3%)	0.035*
16	Provides oxygen according to saturation	12 (40.0%)	19 (63.3%)	0.071
17	Check’s resuscitation responses (pulse, breathing, color, saturation)	13 (43.3%)	22 (73.3%)	0.018*
18	Asks the midwife to start chest compression	27 (90.0%)	30 (100%)	0.237
19	Intubates the baby	12 (40.0%)	21 (70.0%)	0.020*
20	Takes a proper sized tube	24 (80.0%)	30 (100%)	0.024*
21	Intubates in < 30 s with fewer than 3 attempts	18 (60.0%)	28 (93.3%)	0.002*
22	Checks the right position of intubation tube	9 (30.0%)	16 (53.3%)	0.067
23	Gives epinephrine	14 (46.7%)	23 (76.7%)	0.017*
24	Gives the correct amount of epinephrine by the correct route	5 (16.7%)	20 (66.7%)	< 0.001*
25	Gives fluids to correct hypovolemia	4 (13.3%)	21 (70.0%)	< 0.001*
26	Has a plan to arrange treatment extension	4 (13.3%)	17 (56.7%)	< 0.001*
27	Reports the pulse at the beginning	10 (33.3%)	28 (93.3%)	< 0.001*
28	Reports breathing at the beginning	12 (40.0%)	25 (83.3%)	0.001*
29	Reports what she/he has done	25 (83.3%)	23 (76.7%)	0.519
30	Reports the responses of the baby to resuscitation efforts	23 (76.7%)	25 (83.3%)	0.519

*Significant

## Discussion

Neonatal resuscitation is a fundamental skill for nursing students. It is suggested that the efficacy of simulation could be heightened through evidence-based practices [20]. Therefore, Lopreiato and Sawyer suggested adjunctive simulation-based learning in the performance of psychomotor skills [30]. Hence, this study was aimed to investigate the efficacy of the 6-step LSPPDM pedagogy developed by Sawyer et al. [19] for the technical and non-technical skills of nursing students regarding neonatal resuscitation.

The studied cohort was found homogenous in general and resuscitation-related characteristics. The homogeneity among study groups is evident in previous randomized control trials on resuscitation among nursing students [26]. The pre-test score revealed that the resuscitation skill was unsatisfactory among all participants before teaching resuscitation. The most probable explanation was that the nursing students had occasionally been exposed to neonatal resuscitation with infrequent education during their undergraduate studies.

The study demonstrated significant improvement in technical and non-technical skills after education in both groups. Previously, several studies reported that educational intervention had a positive effect on the skills of the providers in the respective field [31–33]. Another major finding of the study was that the post mean difference in technical and non-technical skills was significantly higher in nursing students who received education through LSPPDM pedagogy compared to those who learned through the traditional method. While no research study has yet applied this pedagogy to neonatal resuscitation, an international study utilized the LSPPDM pedagogy for educating the residents’ paracentesis and ultrasound training. The residents reported an increase in confidence and competency in the skill and gave positive feedback [34], thus validated our results regarding the efficacy of LSPPDM pedagogy in the education of neonatal resuscitation.

The results also corroborate with findings by Singhal et al. [10], who performed the Helping Babies Breathe program in two resource-limited areas; Kenya and Pakistan. The study emphasis on active learning and utilized diverse educational strategies with a very similar integration of LSPPDM pedagogy in a diverse group of health professionals. The 31 trainers and 102 learners including a pediatrician, doctors, nurses, midwives, lady health visitors, and lady health workers were selected from both sites. The pre-post assessment of knowledge and skill showed significant gain at cognitive and technical skill levels with an increase in satisfaction and self-efficacy among participants. The findings of the aforementioned study validate the results of the current study that diverse educational strategies following low dose high-frequency have a positive effect on gaining knowledge and skill.

Further, the findings of the current study are in align with another study conducted by Kim and Ahn [17] in Korea, who investigated the effect of the 5-step method versus the traditional method among nursing students. Overall, both interventions improved performance ability. Yet the intervention group performance was significantly higher compared to the control. Despite the difference in strategy and design, the study reported the same results that the 5-step method is suggestively a better approach in learning neonatal resuscitation than the traditional method.

Furthermore, the LSPPDM pedagogy group learned through didactic lectures and proceeded to visual learning through video on neonatal resuscitation. The practice phase was further boosted through clinical exposure, practice under the instructor with an additional self-directed session. Previously, literature published the effectiveness of videos as an effective strategy for improving skill. Pinar, Akalin, and Abay [18] conducted a randomized control trial in Turkey among 46 nursing students to assess the effectiveness of video-assisted simulation versus the traditional lecture method. Their study results were in accordance with our results that although traditional lecture and video-based simulation both are effective strategies for enhancing technical skill, yet, the cumulative effect of simulation with video may potentiate the effect in a positive direction. Additionally, the results are similar to a previous cluster-randomized trial conducted among front-line birth attendants in Uganda. The study reported that the addition of video debriefing in Helping Babies Breathe education could increase the competence and retention of the skill [35].

Moreover, it was observed that nursing students who had learned through LSPPDM pedagogy showed better performance at each step compared to the traditional group. Hence, more than 50% of steps regarding technical and non-technical skills showed a significant difference in performance between the pedagogy and the traditional group. The study results are justified as the efficacy of LSPPDM pedagogy may be attributed to many factors. One difference from the traditional method was the addition of diverse strategies and the other significant difference in contact time (dose). The pedagogy group was repeatedly exposed to learning intervention as compared to the traditional group. Therefore, it is more likely that the improvement in both technical and non-technical skills in the pedagogy group is due to increased contact time. The results are supported by Drake et al. [36] study, who agreed that repeated exposure to learning intervention improves knowledge and skill among participants. Carolan-Olah et al. [37] also suggested the same as the repeated exposure to resuscitation training for a longer period may improve nursing students learning in neonatal resuscitation. Further, the results are in align with Tabangin et al. [38] study, who concluded that frequent practice and exposure to neonatal resuscitation leads towards better acquisition and maintenance of the skill. The study reported that health professionals’ practice frequently showed 2.9 greater odds of passing as compared to those who performed resuscitation less frequently.

Though the study was not intended to measure skill retention, yet the additional maintenance session allowed the participants to frequently practice the skill under self-direction. This may save the time of the instructor and gave the learners a flexible approach to practice their skills without pressure and provided an excellent opportunity to learn through each-others. Moreover, self-directed learning is consistent with adult learning principles that exposed them to active learning with control and flexibility after acknowledging their deficiencies. We believe that the current study adds a significant contribution to the existing literature about the use of the LSPPDM framework during simulation-based nursing education on enhancing skills in neonatal resuscitation specifically in a resource-limited setting.

To the best of our knowledge, this is the first study to investigate the efficacy of LSPPDM pedagogy in the skill learning of neonatal resuscitation among nursing students. The strengths of this study were its design, use of validated tools in the assessment, blinded reviewers, and standardize scenarios in a simulated delivery room situation that enable to make a more objective assessment. Among limitations, it is a single-centered study including only female nursing students, which may limit its generalizability. Since neonatal resuscitation is a team skill that requires multidisciplinary members of the health care team and evaluating the role of each member is important in the resuscitation. The second limitation is that the “do phase” is modified due to the sensitivity of the topic and ethical considerations. This limitation can be justified by the fact was that the nursing students are never allowed to perform practice before license. Thirdly, the skill was assessed in a simulated delivery room and the competency in an actual clinical situation might be challenging. Lastly, the technical and non-technical skills of participants were assessed soon after education, hence the long-term retaining effects were beyond the scope of our study.

Since in today’s health care system, there is a high demand for skilled nursing graduates that respond actively in an emergency. Therefore, this future workforce should be empowered with excellent knowledge and skills, particularly in critical life-saving skills. In Pakistan, nurses are an important member of the health care team and play an active role in the resuscitation team. Hence, their competency in each step may foster their contribution and efficiently performing their role in the team. We believe that this study provides a promising method of addressing neonatal resuscitation education in the nursing curriculum.

## Conclusions

The current study demonstrated the feasibility of LSPPDM pedagogy in the education of neonatal resuscitation among nursing students in resource-limited settings. A significantly positive effect was observed in context with the technical and non-technical skills of nursing students after the intervention. Further trials should be recommended with a large sample size including a diverse group of health professionals and multicentered settings to assess its effectiveness. Moreover, the long-term retention effect after 3–6 months for estimation of real efficacy is warranted.

## Supplementary Information

**Additional file 1.**

## Data Availability

The datasets used and/or analyzed during the current study are available from the corresponding author on reasonable request.

## Abbreviations

LSPPDM pedagogy: Lear, see, practice, prove, do, maintain pedagogy
HBB: Helping Babies Breathe
