# Supplementary material for: Efficacy of pedagogical framework in neonatal resuscitation skill learning in a resource-limited setting: a randomized controlled trial
Source: BMC Med Educ. 2021 Aug 18;21:436. doi: 10.1186/s12909-021-02846-x (PMC8371841; doi:10.1186/s12909-021-02846-x)
Supplement: Supplementary file 1 — Additional file 1. [file 12909_2021_2846_MOESM1_ESM.docx]

**Simulation Scenario**

The scenarios were prepared from the Textbook of Neonatal Resuscitation 7^th^ edition of the American Association of Pediatrics (Nimbalkar et al., 2015a). Here’s below the four simulation scenarios are as follow:

1. A term pregnant lady having a history of gestational diabetes. After the birth of the baby, it was found that the baby is not breathing.
2. An expecting mother having a history of placenta abruption. After birth during the initial evaluation, the baby was found gasping 1minute after birth.
3. A term pregnant woman presenting with a road traffic accident. After birth, it was found that the baby is not crying and having poor muscle tone.
4. The baby has just been born. The baby was not breathing with a heart rate of less than 60 and appear cyanotic.

**ITEMS USED TO ASSESS TECHNICAL SKILLS AND SCORING CRITERIA (Rovamo et al., 2011).**

| **No** | **Items** | **Score 0** | **Score 1** |
| --- | --- | --- | --- |
| 1. | Checks sizes of suction catheters available and if suction equipment is working | Not done | Checked either both or another |
| 2. | Dries the baby and throws wet linen away | Not done | Did either both or another |
| 3. | Checks pulse by palpation or auscultation | Not done | Did either both or another |
| 4. | Stimulates the baby by knocking soles of the feet and rubbing the back | Not done | Did either both or another |
| 5. | Suctions the baby first through mouth and then through the nose | Not done | Did either both or another |
| 6. | Places the head in correct position (nose is at the highest point) | Not done | Done |
| 7. | Takes a proper sized mask | Not done | Done |
| 8. | Starts to ventilate | Not done | Done |
| 9. | Checks that the mask is not leaking | Leaking | No leaking, or only a little leaking |
| 10. | Check’s chest movements | Not done | Chest movement checked |
| 11. | Does not over-expand the lungs | The chest over-expanded, the lung fully expanded on the manikin’s monitor | Proper ventilation |
| 12. | Maintains right ventilation frequency | Ventilation frequency <30/min and long pauses >30 s | Baby ventilated 30–60/min without a pause >30 s |
| 13. | Maintains correct ventilation volume during resuscitation | No chest movement seen | Chest movement seen without a pause >30 s |
| 14. | Uses air at the start | Uses oxygen at the beginning of resuscitation | Resuscitation started with room air |
| 15. | Notice’s cyanosis of the baby | Not done | Pulse oximeter used to follow saturation |
| 16. | Provides oxygen according to saturation | Not Done | Oxygen added according to saturation |
| 17. | Check’s resuscitation responses (pulse, breathing, color, saturation) | Not done | Auscultation or checked pulses, or used pulse oximeter or electrocardiographic monitoring |
| **No** | **Items** | **Score 0** | **Score 1** |
| 18. | Asks the midwife to start chest compression | No plan for continuation of baby’s treatment | Done |
| 20. | Takes a proper sized tube | Did not report breathing at the beginning | Correct size |
| 21. | Intubates in < 30 sec with fewer than 3 attempts | Reported less than half what she/he has done | Done |
| 22. | Checks the right position of intubation tube | Did not mention the responses of the baby, or reported less than half the responses | Auscultation |
| 23. | Gives epinephrine | Epinephrine not given | Gave epinephrine 1 ml/kg intratracheally or 0.3 ml/kg intravenously |
| 24. | Gives the correct amount of epinephrine by the correct route | Epinephrine given intratracheally; small amount | Gave epinephrine 1 ml/kg intratracheally or 0.3 ml/kg intravenously |
| 25. | Gives fluids to correct hypovolemia | No ﬂuid treatment although poor response of the baby to ventilation and epinephrine | Fluid given because of low blood pressure and high pulse level |
| 26. | Has a plan to arrange treatment extension | No plan for continuation of baby’s treatment | Clear plan for the next step of treatment or transport |
| 27. | Reports the pulse at the beginning | Did not report the pulse at the beginning or reported it wrongly | Correct pulse mentioned |
| 28. | Reports breathing at the beginning | Did not report breathing at the beginning | Reported gasps at the beginning |
| 29. | Reports what she/he has done | Reported less than half what she/he had done | Reported ventilation and intubation, oxygen, epinephrine and ﬂuid resuscitation |
| 30. | Reports the responses of the baby to resuscitation efforts | Did not mention the responses of the baby, or reported less than half the responses | Reported responses to ventilation, intubation, oxygen, epinephrine and ﬂuids |

**Assessment of non-technical skills using the nine-item checklist (Rovamo et al., 2011).**

| **No** | **Items** | **1** | **2** | **3** | **4** | **5** |
| --- | --- | --- | --- | --- | --- | --- |
|  | **Task management** |  |  |  |  |  |
| **1.** | Recognizing the situation without delay |  |  |  |  |  |
| **2.** | Continuous evaluation of the patient |  |  |  |  |  |
| **3.** | Prioritizing problems, supporting others |  |  |  |  |  |
| **4.** | Following the algorithm |  |  |  |  |  |
| **5.** | Uninterrupted plans to act |  |  |  |  |  |
|  | **Team working** |  |  |  |  |  |
| **6.** | Leadership, coordinating activities |  |  |  |  |  |
| **7.** | Communication |  |  |  |  |  |
|  | **Situation awareness** |  |  |  |  |  |
| **8.** | Vigilance and anticipation |  |  |  |  |  |
| **9.** | Adequate medical knowledge |  |  |  |  |  |
